# Predictors of early high-level response to bimekizumab in patients with moderate-to-severe plaque psoriasis: a real-world cohort study^؆^

**DOI:** 10.1016/j.abd.2026.501353

**Published:** 2026-04-22

**Authors:** Burhan Engin, Yusuf Demir, Gurbuz Yildirim, Fatma Zehra Diken, Bengusu Yildiz, Zekayi Kutlubay, Server Serdaroglu

**Affiliations:** Department of Dermatology, Faculty of Medicine, Istanbul University-Cerrahpasa, Istanbul, Turkey

Dear Editor,

Bimekizumab is a highly effective therapeutic option in the management of psoriasis; however, treatment response remains heterogeneous in real-world practice, and baseline predictors of high-level response have not been well characterized in routine clinical settings.^1^, ^2^, ^3^ Accordingly, this study aimed to identify baseline characteristics predictive of achieving a Psoriasis Area and Severity Index (PASI) -90 response at week-16 in patients with moderate-to-severe psoriasis receiving bimekizumab.

Advances in the management of moderate-to-severe psoriasis have been driven by biologic therapies targeting the IL-23/IL-17 axis, a pathway central to the disease's immunopathogenesis.^4^ Bimekizumab, a humanized monoclonal antibody designed for the dual neutralization of IL-17A and IL-17 F, has demonstrated superior efficacy in phase 3 trials over selective IL-17A or TNF-α inhibitors.^1^, ^2^, ^3^ The dual-inhibition strategy is to achieve a more comprehensive suppression of psoriatic inflammation by targeting the distinct pathogenic roles of both IL-17 isoforms.^5^

The expanding therapeutic landscape for psoriasis presents a critical challenge in selecting the optimal biologic for individual patients. This challenge is compounded by the substantial response heterogeneity observed in real-world practice. Identifying baseline factors predictive of therapeutic success is therefore crucial for advancing personalized medicine. Although factors such as baseline disease severity, body weight, and prior biologic exposure have been implicated in the response to various biologics,^6^ robust predictors for bimekizumab in routine clinical care have yet to be defined. Accordingly, this study aimed to identify baseline characteristics predictive of achieving a PASI-90 response at week-16 in patients with moderate-to-severe psoriasis receiving bimekizumab.

We conducted a retrospective cohort study of adults with moderate-to-severe chronic plaque psoriasis who initiated bimekizumab between December 2024 and June 2025 at a single tertiary center. The primary endpoint was PASI-90 response at week-16. We extracted baseline demographics, disease characteristics, difficult-to-treat site involvement, and prior treatment history from electronic medical records. The severity of the disease was categorised according to the patient's PASI score at the beginning of the study. This was consistent with the severity thresholds established for clinical practice and therapeutic guidelines: moderate psoriasis (PASI 10–20) and severe psoriasis (PASI > 20).

All statistical analyses were conducted using *R* version 4.3.0. Baseline characteristics were compared between patients who achieved a PASI-90 response (responders) and those who did not (non-responders). Variables with a potential association in the univariate analysis (p < 0.20) were included in a multivariate logistic regression model. A final, parsimonious model was then derived via a backward elimination procedure, retaining only independent predictors with a significance level of p < 0.05.

The final analysis included a cohort of 102 patients. Baseline clinical and demographic characteristics are presented in Table 1, while characteristics of responders and non-responders are presented in Table 2. The cohort was predominantly male (63.7%) with a mean age of 47.2-years. Patients presented with established, moderate-to-severe disease, as reflected by a mean disease duration of 14.6-years and a mean baseline PASI score of 16.8 (range: 10.2–38.4). The majority of patients had mild-to-moderate disease severity (baseline PASI 0–20: n = 68, 66.7%), while 34 patients (33.3%) had severe disease (baseline PASI > 20). A majority of patients (54.9%) were biologic-experienced. At the primary endpoint of week-16, a PASI-90 response was achieved by 80 of 102 patients (78.4%).

**Table 1:** Baseline characteristics of study population (n = 102).

Characteristic	Value
**Demographics**	
Age, mean ± SD, years	47.2 ± 13.8
Male sex, n (%)	65 (63.7)
Body mass index, mean ± SD, kg/m²	28.1 ± 5.4
Current smoking, n (%)	48 (47.1)
**Disease Characteristics**	
Disease duration, mean ± SD, years	14.6 ± 9.2
Age of disease onset, mean ± SD, years	32.6 ± 12.1
Baseline PASI score, mean ± SD	16.8 ± 8.7
PASI 0–20 (mild-moderate), n (%)	68 (66.7)
PASI > 20 (severe), n (%)	34 (33.3)
Family history of psoriasis, n (%)	43 (42.2)
Psoriatic arthritis, n (%)	31 (30.4)
**Special Area Involvement, n (%)**	
Scalp	53 (52.0)
Nail	44 (43.1)
Palmoplantar	29 (28.4)
Genital	22 (21.6)
Face	24 (23.5)
**Prior Treatment History**	
Biologic-naive, n (%)	46 (45.1)
1 prior biologic, n (%)	32 (31.4)
≥ 2 prior biologics, n (%)	24 (23.5)
**Prior Biologic Classes (n = 56), n (%)**	
Anti-TNF	25 (44.6)
Anti-IL-17	19 (33.9)
Anti-IL-23	25 (44.6)
Anti-IL-12/23	10 (17.9)
**Comorbidities, n (%)**	
Any comorbidity	77 (75.5)
Hypertension	36 (35.3)
Diabetes mellitus	19 (18.6)
Dyslipidemia	17 (16.7)
Psychiatric disorders	24 (23.5)
Respiratory diseases	17 (16.7)

**Table 2:** Baseline characteristics by PASI-90 response status at week-16.

Characteristic	PASI90 Responders (n = 80)	Non-responders (n = 22)	p-value
**Demographics**			
Age, mean ± SD, years	44.2 ± 12.1	52.8 ± 14.6	**0.006**
Male sex, n (%)	52 (65.0)	13 (59.1)	0.61
BMI, mean ± SD, kg/m^2^	28.0 ± 5.5	28.4 ± 5.2	0.76
**Disease Characteristics**			
Disease duration, mean ± SD, years	14.3 ± 9.3	15.7 ± 8.9	0.53
Age of disease onset, mean ± SD, years	29.9 ± 11.2	37.1 ± 13.5	**0.012**
Baseline PASI, mean ± SD	13.4 ± 6.8	18.9 ± 9.2	**0.003**
**Special Area Involvement, n (%)**			
Scalp	41 (51.2)	12 (54.5)	0.78
Nail	23 (28.8)	12 (54.5)	**0.01**
Palmoplantar	22 (27.5)	7 (31.8)	0.69
Genital	17 (21.2)	5 (22.7)	0.88
Face	18 (22.5)	6 (27.3)	0.64
**Treatment History**			
Biologic-naive, n (%)	41 (51.2)	6 (27.3)	**0.03**
1 prior biologic, n (%)	24 (30.0)	8 (36.4)	0.56
≥2 prior biologics, n (%)	15 (18.8)	8 (36.4)	0.08
**Comorbidities, n (%)**			
Any comorbidity	58 (72.5)	19 (86.4)	0.17
Hypertension	25 (31.2)	11 (50.0)	0.10
Diabetes mellitus	13 (16.2)	6 (27.3)	0.24
Psychiatric disorders	17 (21.2)	7 (31.8)	0.30
Psoriatic arthritis	23 (28.8)	8 (36.4)	0.48

Continuous variables compared using Student's *t*-test or Mann-Whitney *U*-test.

Categorical variables compared using χ² or Fisher's exact test.

Multivariate logistic regression analysis identified three independent baseline predictors of a PASI-90 response at week-16 (Table 3, Fig. 1). A biologic-naive status was positively associated with the outcome (Odds Ratio [OR = 3.24]; p = 0.008), whereas both a higher baseline PASI score (OR = 0.94; p = 0.021) and the presence of nail involvement (OR = 0.18; p = 0.001) were inversely associated with achieving the endpoint. The final predictive model yielded an Area Under the receiver operating Characteristic Curve (AUC) of 0.78.

**Table 3:** Univariate and multivariate logistic regression analysis for PASI-90 response at week-16.

Variable	Univariate Analysis		Multivariate Analysis	
	OR (95% CI)	p-value	OR (95% CI)	p-value
**Demographics**				
Age ≤ 45-years (vs. > 45)	2.43 (1.21–4.88)	**0.012**	2.12 (0.95–4.73)	0.067
Male sex	1.28 (0.64–2.55)	0.481	‒	‒
BMI (per kg/m^2^)	0.98 (0.92–1.05)	0.625	‒	‒
Current smoking	0.86 (0.43–1.72)	0.671	‒	‒
**Disease Characteristics**				
Disease duration (per year)	0.98 (0.94–1.03)	0.532	‒	‒
Baseline PASI (per unit)	0.91 (0.86‒0.96)	**0.001**	0.94 (0.89‒0.98)	**0.021**
Family history of psoriasis	0.89 (0.44–1.78)	0.743	‒	‒
Psoriatic arthritis	0.69 (0.33–1.44)	0.324	‒	‒
**Special Area Involvement**				
Scalp	0.88 (0.44–1.76)	0.721	‒	‒
Nail	0.34 (0.16‒0.72)	**0.005**	0.18 (0.06‒0.47)	**0.001**
Palmoplantar	0.82 (0.37–1.81)	0.623	‒	‒
Genital	0.91 (0.38–2.19)	0.834	‒	‒
Face	0.77 (0.33–1.79)	0.544	‒	‒
**Treatment History**				
Biologic-naive	2.78 (1.35–5.72)	**0.006**	3.24 (1.42–7.85)	**0.008**
Number of prior biologics				
0 (reference)	1.00	‒	‒	‒
1	0.44 (0.20‒0.97)	**0.041**	‒	‒
≥ 2	0.27 (0.11‒0.65)	**0.003**	‒	‒
**Comorbidities**				
Any comorbidity	0.42 (0.16–1.11)	0.081	0.58 (0.19–1.76)	0.337
Hypertension	0.46 (0.21‒0.98)	**0.045**	0.71 (0.29–1.74)	0.451
Diabetes mellitus	0.51 (0.20–1.31)	0.164	‒	‒
Dyslipidemia	0.74 (0.28–1.95)	0.541	‒	‒
Psychiatric disorders	0.58 (0.24–1.39)	0.223	‒	‒

OR, Odds Ratio; CI, Confidence Interval; BMI, Body Mass Index.

Variables with p < 0.20 in univariate analysis were entered into the multivariate model. Final model was determined using backward stepwise selection with retention threshold p < 0.10.

OR < 1 indicates decreased odds of achieving PASI90 response; OR > 1 indicates increased odds of achieving PASI90 response.

Model performance: Area under the curve = 0.78 (95% CI 0.68‒0.88); Hosmer-Lemeshow goodness-of-fit test p = 0.42.

**Fig. 1:**
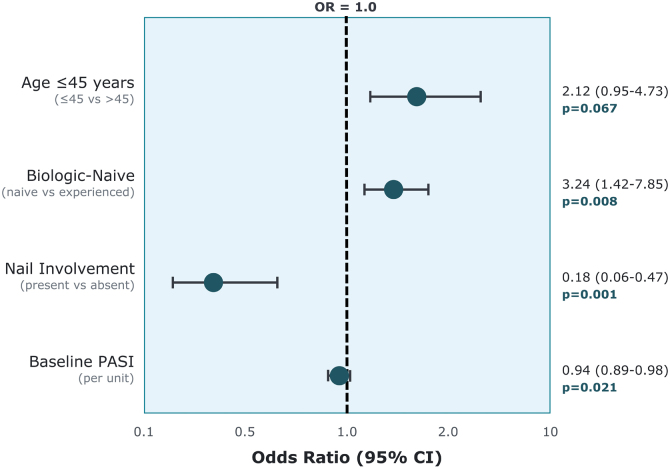
Forest plot showing multivariate predictors of PASI-90 response at week-16. OR > 1 indicates favorable factor; OR < 1 indicates unfavorable factor.

Superior efficacy in biologic-naive patients with psoriasis carries substantial clinical implications. These findings underscore that the first biologic therapy represents the optimal opportunity for achieving robust and durable treatment response. This therapeutic window progressively narrows with each subsequent line of therapy ‒ a phenomenon attributable to two key mechanisms: the selection of inherently treatment-resistant disease phenotypes and the emergence of neutralizing anti-drug antibodies.^7^ Our data advocate for the strategic deployment of highly effective biologics early in the treatment algorithm to maximize long-term clinical outcomes. Physicians should prioritize positioning the most potent therapeutic agents as first-line treatment, rather than reserving these medications for treatment-refractory disease.

The inverse relationship between baseline PASI score and treatment success is also an important finding. For each one-unit increase in baseline PASI, the odds of achieving PASI-90 decreased by 6% (OR = 0.94, 95% CI 0.89–0.98). This result is consistent with reports for other highly effective biologics,^8^ suggesting that a greater inflammatory burden may indicate a more persistent disease state that is difficult to resolve completely. This observation supports the current clinical strategy of early and effective intervention.

Nail involvement emerged as the strongest negative predictor of achieving near-complete skin clearance, highlighting a major therapeutic challenge. The nail unit presents significant treatment barriers due to its unique anatomical structure and specialized immune environment, which creates a drug-resistant site with limited drug penetration and persistent inflammatory cells.^9^ This finding aligns with clinical observations showing nail psoriasis takes much longer to clear than skin lesions, even with highly effective biologics like bimekizumab.^10^ The results show the need for realistic treatment expectations and potentially more intensive therapeutic approaches in patients with baseline nail involvement.

Findings from the initial 16-week treatment period underscore the importance of managing patient expectations, particularly for individuals with nail psoriasis. Clinicians should counsel patients about the differential response rates: rapid skin clearance remains achievable, while nail disease resolution follows a more gradual trajectory requiring sustained treatment. Notably, traditional metabolic factors, including BMI and common comorbidities, did not emerge as significant predictors of response in this analysis. Bimekizumab appears to maintain robust efficacy across diverse patient subgroups. The consistent efficacy across different demographic and clinical profiles supports the broad applicability of our predictive model in routine practice.

In this real-world analysis of patients (n = 102) with moderate-to-severe psoriasis, three baseline factors were identified as independent predictors of an early high-level response to bimekizumab: prior exposure to biologics, severity of disease, and nail involvement. Patients without prior biologic exposure demonstrated superior treatment outcomes. Conversely, elevated baseline PASI scores and the presence of nail involvement were identified as negative predictors for achieving a PASI-90 response at the 16-week timepoint. The stratification of patients by expected treatment response, optimization of therapeutic choices, and establishment of realistic goals are made possible by these predictive factors. A practical framework is thus provided for the individualization of bimekizumab therapy and for guiding patient counseling regarding anticipated outcomes in routine practice.

## ORCID ID

Yusuf Demir: 0000-0003-3307-5917

Gurbuz Yildirim: 0000-0003-4195-1825

Fatma Zehra Diken: 0009-0009-8855-4617

Bengusu Yildiz: 0009-0006-9941-241X

Zekayi Kutlubay: 0000-0003-0809-1624

Server Serdaroglu: 0000-0003-2239-9430

## Data availability statement

The data that support the findings of this study are available from the corresponding author upon reasonable request.

## Financial support

None declared.

## Authors' contributions

Burhan Engin: Contributed to conceptualization, methodology, and supervision.

Yusuf Demir: Was responsible for data curation, investigation, and writing the original draft.

Gurbuz Yildirim: Performed data curation, formal analysis, and contributed to writing-review and editing.

Fatma Zehra Diken: Handled investigation, data curation, and visualization.

Bengusu Yildiz: Assisted with data curation and investigation.

Zekayi Kutlubay: Contributed to methodology, resources, and writing-review and editing.

Server Serdaroglu: Provided supervision, project administration, and contributed to writing-review and editing.

## Research data availability

The entire dataset supporting the results of this study was published in this article.

## Conflicts of interest

None declared.
